# Language and gender: Computerized text analyses predict gender ratios from organizational descriptions

**DOI:** 10.3389/fpsyg.2022.1020614

**Published:** 2023-01-09

**Authors:** Lotta Stille, Sverker Sikström, Anna Lindqvist, Emma A. Renström, Marie Gustafsson Sendén

**Affiliations:** ^1^Department of Psychology, Lund University, Lund, Sweden; ^2^Department of Psychology, Kristianstad University, Kristianstad, Sweden; ^3^Department of Psychology, Stockholm University, Stockholm, Sweden

**Keywords:** organizational descriptions, gender segregation, job market, perceived fit, natural language processing

## Abstract

Previous research has shown that language in job adverts implicitly communicates gender stereotypes, which, in turn, influence employees’ perceived fit with the job. In this way, language both reflects and maintains a gender segregated job market. The aim of this study was to test whether, and how, language in organizational descriptions reflects gender segregation in the organizations by the use of computational text analyses. We analyzed large Swedish companies’ organizational descriptions from LinkedIn (*N* = 409), testing whether the language in the organizational descriptions is associated with the organizations’ employee gender ratio, and how organizational descriptions for organizations with a majority of women and men employees differ. The statistical analyses showed that language in the organizational descriptions predicted the employee gender ratio in organizations well. Word clouds depicting words that differentiate between organizations with a majority of women and men employees showed that the language of organizations with a higher percentage of women employees was characterized by a local focus and emphasis on within-organizations relations, whereas the language of organizations with a higher percentage of men employees was characterized by an international focus and emphasis on sales and customer relations. These results imply that the language in organizational descriptions reflects gender segregation and stereotypes that women are associated with local and men with global workplaces. As language communicates subtle signals in regards to what potential candidate is most sought after in recruitment situations, differences in organizational descriptions can hinder underrepresented gender groups to apply to these jobs. As a consequence, such practices may contribute to gender segregation on the job market.

## Introduction

The aim of this study was to test whether, and how, language in organizational descriptions reflects gender segregation in the organizations, by the use of different computational methods to investigate the relationship between organizational descriptions and the employee gender ratios of the examined organizations. Previous research in this area has mainly examined language in job requirement descriptions and job adverts (Gaucher et al., 2011; Pietraszkiewicz et al., 2018). However, little is known about the language used in more general texts describing organizations. Organizational descriptions often accompany job adverts or stand alone to let people assess organizations they may be interested in working for. On the Internet, these texts are often found under the heading “About us.” In contrast to job adverts, which usually focus on information about the specific position, descriptions of organizations are typically more general and comprehensive, thereby including information relevant to all employees in the organization, regardless of their position. Organizational descriptions tend to focus on attributes of the organization and may include the line of work the organization is involved with; origins and background of the organization; government of the organization; vision, objectives, goals, and previous accomplishments of the organization; geographical and demographic information related to the organization; various policies of the organization (e.g., career development and diversity); qualifications and competencies within the organization (Backhaus, 2004).

Language in job adverts has been shown to reflect the gender distribution in a profession (Gaucher et al., 2011; Pietraszkiewicz et al., 2018). Adverts for women-dominated jobs include more words related to stereotypical feminine attributes, i.e., related to communion (e.g., caring, understanding, and compassionate), whereas adverts for men-dominated jobs include more words related to stereotypical masculine attributes, i.e., related to agency (e.g., confident, determined, and ambitious; Abele and Wojciszke, 2014). Such differences affect whether women and men perceive a fit between themselves and the job position (Bosak and Sczesny, 2008; García et al., 2008). The present study investigates if these findings can be extended from job adverts to organizational descriptions, and thereby examines whether organizational descriptions reflect the gender distribution in the organization. The focus is organizational descriptions on LinkedIn, an online platform used for professional networking where employers post job openings and job applicants can post their CVs.

Gender segregation on the job market is common (Blackburn and Jarman, 2006). For example, women dominate the health care sector and men the technical and engineering professions (e.g., Watt, 2010; Croft et al., 2015; Tellhed et al., 2017). Even though Sweden is one of the most gender equal countries in the world (Zahidi et al., 2018), with a nearly equal employment rate for women (84.5%) and men (89.4%), the job market is still gender segregated, based on legal gender (Statistics Sweden, 2018). Gender segregation appears both between different industries and within specific organizations. The professions of women are often lower in status, lower in salary, and there are fewer women in senior positions as compared with men (Statistics Sweden, 2018).

According to social role theory (Eagly, 1987), gender stereotypes and a gender segregated job market are deeply intertwined. More specifically, observing women and men working in different industries and positions, influence perceptions of personality traits corresponding to the competencies needed in those professions (Eagly and Wood, 2012). Thus, when women perform the role of homemaker or in professions related to the domestic role in jobs, they are presumed to be communal, whereas, when men perform tasks in the job market associated with higher status, they are presumed to be more agentic (Eagly and Wood, 2012; Gustafsson Sendén et al., 2019; Eagly et al., 2020). In this way, a better balanced gender equality between women and men in the job market would theoretically decrease gender stereotypes of women and men.

To counteract gender segregation on the job market, both legal and affirmative actions are needed. For example, gender discrimination in job adverts is prohibited in Sweden according to Swedish law, yet research suggests that recruitment situations still contribute to maintaining gender segregation (Bygren, 2013). Through wordings regarding characteristics mainly associated with either femininity or masculinity (e.g., related to communion or agency), job adverts and organizational descriptions may still implicitly address a particular category of applicants (Gaucher et al., 2011).

Language implicitly conveys norms (e.g., Weatherall, 2002), and specific choices of words can reflect stereotypes relating to gender (e.g., Pietraszkiewicz et al., 2018). Because language affects individuals’ perception of themselves and the world, as well as how they convey perceptions of reality (Fiedler, 2008; Beukeboom and Burgers, 2019), language can contribute to maintaining a segregated job market. Some job titles and wordings are explicitly gendered, for example chairman, fireman, and policeman, which may lead to men perceiving a better fit with the job. Masculine generics have also been used in job adverts (Bem and Bem, 1973), meaning that a potential employee was referred to as *he*. This is no longer the case. However, in grammatically and linguistically gendered languages, job titles are still more often described with the generic masculine form instead of a gender-neutral equivalent (Sczesny et al., 2016). Masculine generics influence perceptions that a man better matches the position than a woman applicant (Stout and Dasgupta, 2011).

Other wordings are more implicit. For example, job adverts for women-dominated professions and positions have been found to use a more communal wording (e.g., supportive and kind), whereas adverts for men-dominated professions and positions use a more agentic wording (e.g., competitive, dominant; Gaucher et al., 2011; Askehave and Zethsen, 2014; Ulfsdotter Eriksson and Backman, 2014; Pietraszkiewicz et al., 2018). Traits described and requested in job ads, such as *understanding* – communion, and *assertive –* agency, can activate gender related stereotypes regarding who is best suited for the job (Abele, 2003; Gaucher et al., 2011). Language can therefore contribute to reinforcing, as well as counteracting, existing gender segregation, by affecting both who is seen as a fit with, and who feels motivated to apply for the job (e.g., Trömel-Plötz, 1982; Horvath et al., 2015).

According to the lack of fit model (Heilman, 1995), a match or a mismatch between gender stereotype characteristics and perceived professional role requirements influence judgments in recruitment settings, for both possible applicants and recruiters. A match enhances expectations of role performance success, while a mismatch brings expectations of performance failure (Heilman, 2012). For applicants, a lack of fit might decrease motivation to apply and belief in having the right competences; and, for recruiters, a lack of fit might lead to beliefs that the applicant lacks the capability needed to do well in this position and therefore make them refrain from hiring that person (Heilman and Caleo, 2018).

Perceived fit in recruitment contexts can be applied both to the individual’s fit with a specific job, person-job fit, as well as to the individual’s fit with an organization as a whole, person-organization fit (Kristof-Brown et al., 2005). From an applicant’s perspective, perceived person-organization fit between oneself and organization can predict the appeal of the organization and of a new job, as well as intentions of pursuing it (Dineen et al., 2002; Chapman et al., 2005). Person-organization fit occurs when employer and/or employee “provides what the other needs, when they share similar fundamental characteristics, or both” (Kristof, 1996, pp. 4–5). Hence, it is important that values and principles of the organization align with those of the potential employee, and that employee and job/organization do not experience a mismatch. A challenge for organizations is, thus, to communicate organizational values without excluding qualified presumptive candidates (Kristof-Brown et al., 2005; Klysing et al., 2021). The organizational description allows organizations to brand themselves and thereby let presumptive employees assess their person-organization fit (Carless, 2005). Furthermore, the organizational description is a way for organizations to broaden their workforce. Organizations might need to change their word choices to attract employees that, due to gender stereotypes, are not typical to the organization (Klysing et al., 2021).

Recruitment gives real life opportunities of increasing gender equality in professions and organizations. It is therefore important to examine whether language in job adverts and organizational descriptions reinforce and perpetuate gender segregation in the job market. Before, organizations used print advertisements and agencies to reach job candidates (Blacksmith and Poeppelman, 2014). Today, the Internet and social media are the principal way for both employers and prospective employees to market themselves (LinkedIn, 2015). This new way of recruitment allows organizations to target not just active job candidates, i.e., currently unemployed individuals actively seeking jobs, but also passive job candidates, i.e., currently employed individuals not actively seeking new jobs, but who would consider changing jobs if they perceive a better match between themselves and the organizations (Joos, 2008).

In the present study, organizational descriptions from large Swedish organizations published on LinkedIn were used for the analyses. LinkedIn is an online networking service that has over 700 million users, and 9 million corporate accounts in more than 200 countries worldwide (Awan, 2017; LinkedIn, 2019). As of October 2018, LinkedIn had 3.5 million Swedish users, i.e., 35% of the population (The Swedish Internet Foundation, 2018). An advantage of using LinkedIn, is that the organizations present themselves toward potential applicants also when they do not have ongoing recruitment processes. Using observational data, the present study thus examines the relationship between different language and gender distribution of work organizations.

The aim of this study was to test whether, and how, language in organizational descriptions is associated with the ratio of women and men in the organizations, using computational text analysis. In light of previous research, there is a need for studies that illuminate the language organizations use to describe themselves, and that specify in which ways language might differ between organizations with different gender ratios. We used legal gender as measurement of gender distribution within the organizations, therefore we could not include other genders than women/men.

Computerized text analyses were used to examine the association between texts, i.e., organizational descriptions downloaded from LinkedIn, and a numerical value, i.e., employee gender ratios of the examined organizations. The text analyses were completed by Latent Semantic Analysis (Landauer et al., 2007) and Bidirectional Encoder Representations from Transformers (BERT; Devlin et al., 2019), which are two completely data-driven methods. Pearson correlations between linguistic measures and statistical measures of gender ratio were computed with the aim of answering the following questions:

1. Is the language in organizational descriptions associated with the gender ratios in organizations?
2. How do content and word choice differ between organizational descriptions for organizations with a majority of women and a majority of men employees?

## Materials and methods

### Sample and coding

#### Inclusion and exclusion criteria

**Figure 1:**
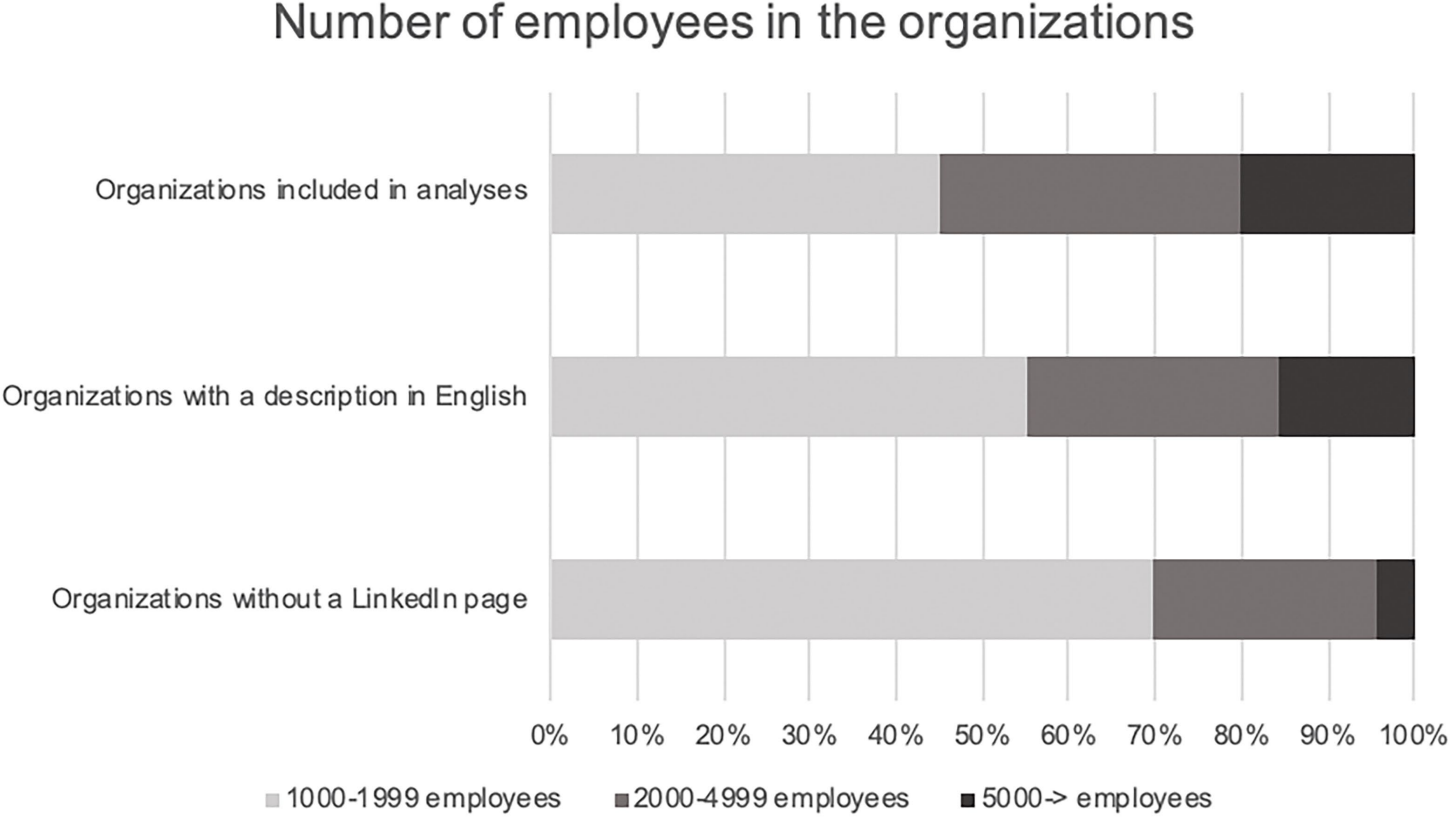
Sizes of included and excluded organizations.

**Figure 2:**
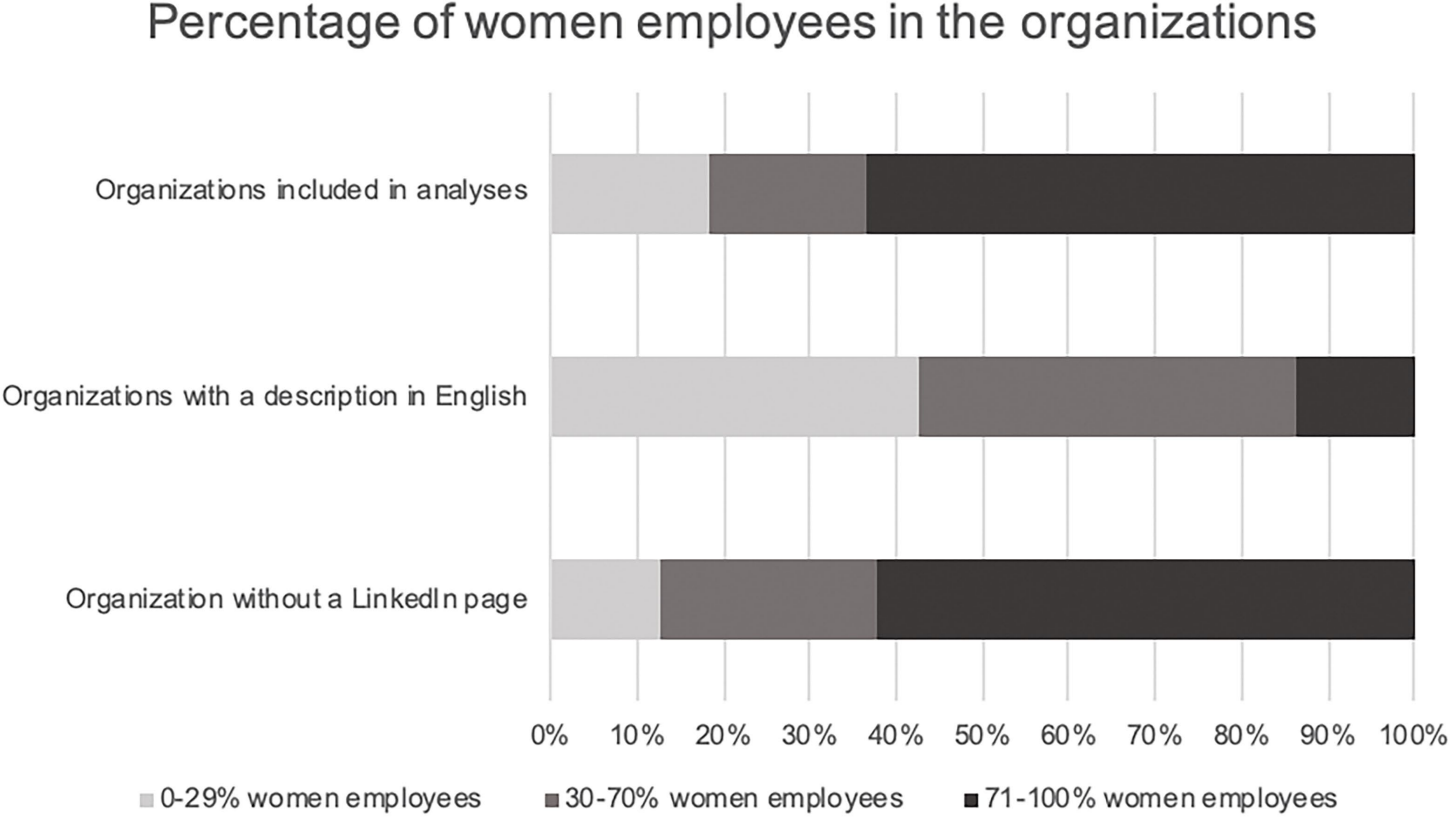
Gender ratios in included and excluded organizations.

All private and public Swedish organizations (*N* = 564) with more than 1,000 employees in Sweden were selected for the study, as these medium to large sized organizations were probable to market themselves on LinkedIn. *Private* organizations (*n* = 256, 45%) and *public* organizations (*n* = 308, 55%) were selected based on statistics from Statistics Sweden.^1^ The analyzed texts were downloaded from LinkedIn under the template headline “About us.” Sixty-six (12%) of the organizations had no LinkedIn-page or no organizational description, and were therefore excluded from the analyses. Also, 89 (16%) organizations that only had English organizational descriptions were excluded from the analyses. The final sample thus consisted of 409 Swedish organizational descriptions. Of these, 278 (68%) were public and 131 (32%) were private. The total number of words in the sample, after excluding organizations that were not included, was 54,383. The mean number of words in the texts were *N* = 133 (SD = 65) and the mean sentence length was *N* = 9.2 words (SD = 2.8). Neither the number of words in the texts, nor the sentence length, correlated with the gender ratios. Figures 1, 2 show an overview of the included and excluded organizations.

#### Basis for semantic analyses

To make sure that the analysis is not dependent on specific model implementations, we analyzed our data using two distinctly different models: Latent Semantic Analysis, LSA (e.g., Landauer, 1999; Kjell et al., 2018) and the Bidirectional Encoder Representations from Transformers, BERT (Devlin et al., 2019). LSA is a bag-of-word model that creates semantic representations of words, but cannot capture the grammatical structure of the data. BERT is a deep neural network that uses transformers to quantify the meaning of texts. This model can capture grammatical structure of text data. The results of the LSA model are presented in the main texts, whereas the results of the BERT model are found in the Appendix.

The overall aim was to study gender differences in organizational descriptions. For methodological details, and how the models can be used to predict a numerical value and optimization, we refer to other articles (e.g., Landauer, 1999; Kjell et al., 2018) and the Appendix. The data analysis was conducted in SemanticExcel, which is an online statistical software application that analyzes texts that allows calling the LSA model and the BERT model that is described elsewhere (e.g., Sikström et al., 2020; Sikström and Garcia, 2020). Finally, the source code for the LSA analysis is available on Github.^2^

## Results

### Semantic analyses

To answer the first research question regarding whether organizational descriptions are associated with the gender ratio (i.e., women/men) in the organizations, we used the linear regression method described in detail in Kjell et al. (2018) and the Appendix. In this, the relationship between texts (i.e., from LinkedIn) and a numerical variable (i.e., gender ratio of women/men) was analyzed using the semantic representations of the texts as generated from LSA or BERT as input.

Table 1 shows examples of LinkedIn texts and their associated observed gender ratios and ratios predicted from the regression model (based on legal gender; percentage women).

**Table 1:** Observed and predicted gender ratios.

LinkedIn text	% Women employees	
	Observed	Predicted
**At BAUHAUS,** we always strive for there to be much more to go for. That is why we want employees who know a little more than most. We want to be a leader in our field, be able to offer the industry’s largest selection and provide efficient customer service. That is why our employees are important, because only by having motivated and committed employees can BAUHAUS take a leading position. At BAUHAUS, we give all employees the opportunity to show what they can do by providing space for their own initiatives. We believe that professional challenges, development opportunities and the opportunity for personal responsibility provide employees who are the big difference in everyday work. It is not decisive what background our employees have, but a background as a craftsman or with experience from the retail trade would be good. We first and foremost recruit staff for leading positions internally. Therefore, we can offer our employees development and career opportunities both in Sweden and abroad.	46	64
**Arbetsförmedlingen** is Sweden’s largest mediator of jobs. Our most important task is to bring together those who need to hire with those who are looking for work. By creating meeting places between employers and jobseekers, we contribute to a well-functioning labor market. As an authority, we receive our assignment from the Riksdag and the government. We are about 14,000 who work at the Swedish Public Employment Service in a number of different professional areas. Most of us are job brokers, but we are also psychologists, IT specialists, investigators, occupational therapists, social workers and more.	66	59
**Burlöv municipality** is life and movement between Lund and Malmö, the creative meeting place for housing, business, development and culture. For us, it is important that our nearly 18,000 citizens, approximately 1,200 employees and visitors feel welcome and safe. We provide high quality service, always with the citizens and the business community at the center. Our values for treatment, trust, participation, equal treatment are important to us in our work. Burlöv is also close! The geographical location provides excellent commuting opportunities by bike, train and bus. Do you want to be part of our journey and continue to develop the municipality of Burlöv?	77	75

Examples of LinkedIn texts (translated into English using Google Translate) with observed and predicted gender ratios based on legal gender.

The Pearson correlations between observed and predicted values were computed to indicate how well the LinkedIn texts predict the observed gender ratio based on legal gender. If the organizational texts predict the proportion of women exactly, the correlation is 1.

The Pearson correlation between the observed and predicted binary employee gender ratio of the organizations was significantly larger than zero for the LSA model (*r* = 0.65, *p* < 0.0001; *r*^2^ = 42%, Mean Absolute Error = 12.3%, *n* = 409, see Appendix for the results of the BERT model). The explained variance in the text set indicated a large effect. Thus, LinkedIn texts are associated with the gender ratio of employees in an organization.

A possibility is that our results simply could be explained by whether the organization was a private company, or a part of the Swedish public sector. To deal with this issue, we added a binary variable coding for private/public organization as a covariate to the multiple linear regression model based on the LSA representations. The results showed that the gender ratio still could be predicted with a reasonably high Pearson correlation (*r* = 0.45, *p* < 0.001, *r*^2^ = 20%, Mean Absolute Error = 12.6%, see Appendix for the results of the BERT model), although the Pearson correlation was somewhat lower compared to when the analysis without this covariate.

### Content analyses of organizational descriptions

To answer the second research question, i.e., *how* organizational descriptions for organizations with a majority of women or men employees differ from each other, the words from the LinkedIn texts were further analyzed semantically and illustrated using word clouds.

The closeness of two semantic representations (i.e., words or whole texts) is a measure of their semantic similarity. This can be calculated by the cosine of the angle between the two semantic representations in the semantic space. A high semantic similarity score signifies a high semantic similarity between two semantic representations, and a low score indicates that the two semantic representations are unrelated in meaning. For example, the semantic similarity between *organization* and *employee* is higher than that between *organization* and *balloon*. In this way, similarities and differences between texts or words can be measured with numbers. This, in turn, enables standard statistical methods, such as *t*-tests to test how words or text sets differ.

**Figure 3:**
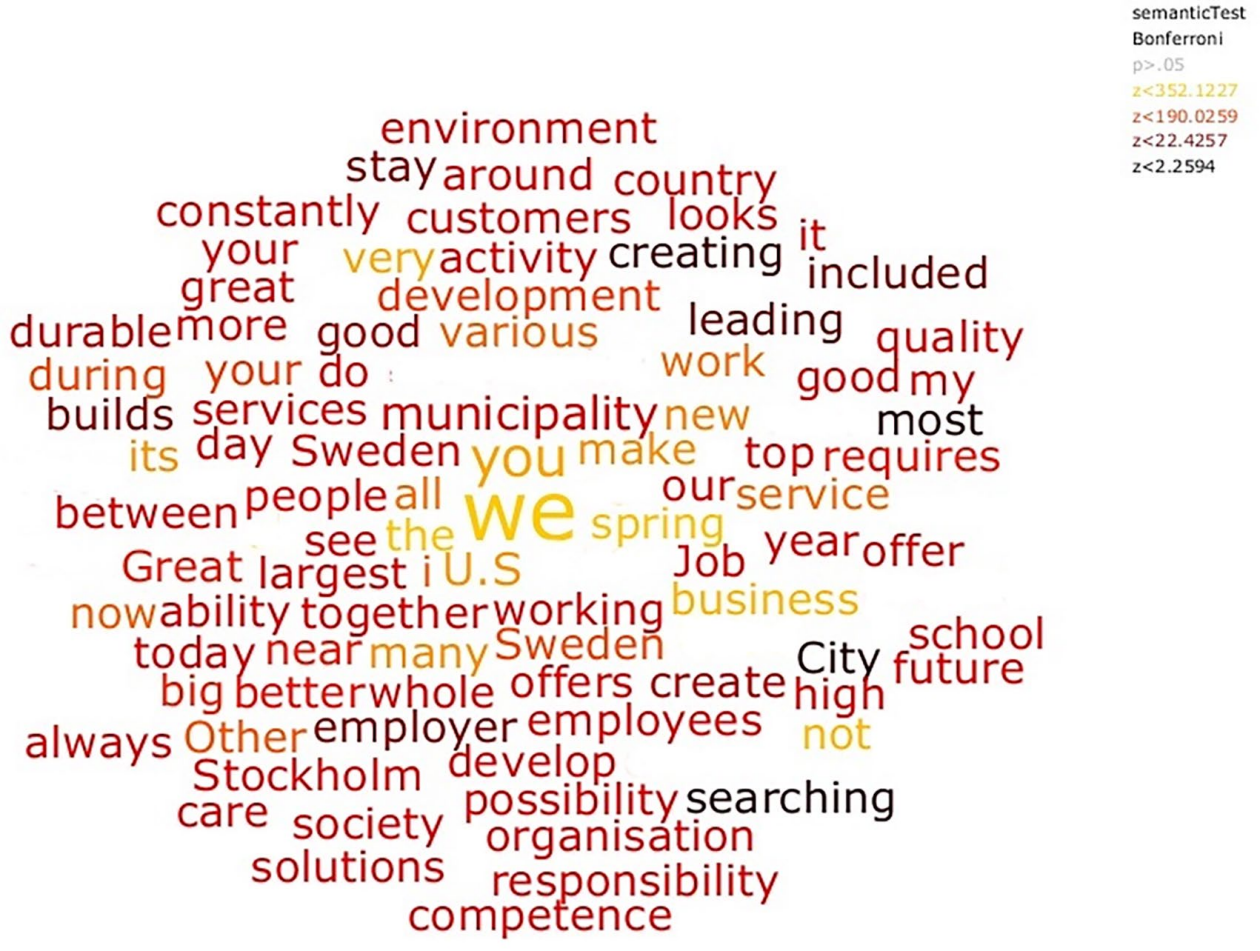
The most indicative words from all examined LinkedIn descriptions contrasted with how the words are used in the Swedish version of Google n-gram database and tested for significance by a semantic *t*-test.

**Figure 4:**
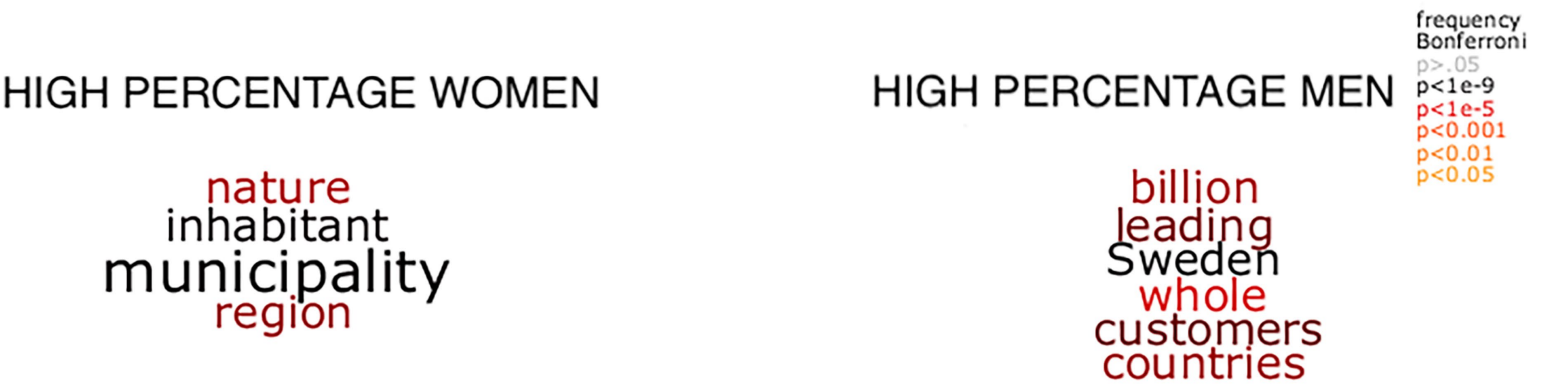
Word clouds depicting words indicative of a high percentage of women and men as significantly tested by chi-square tests.

**Figure 5:**
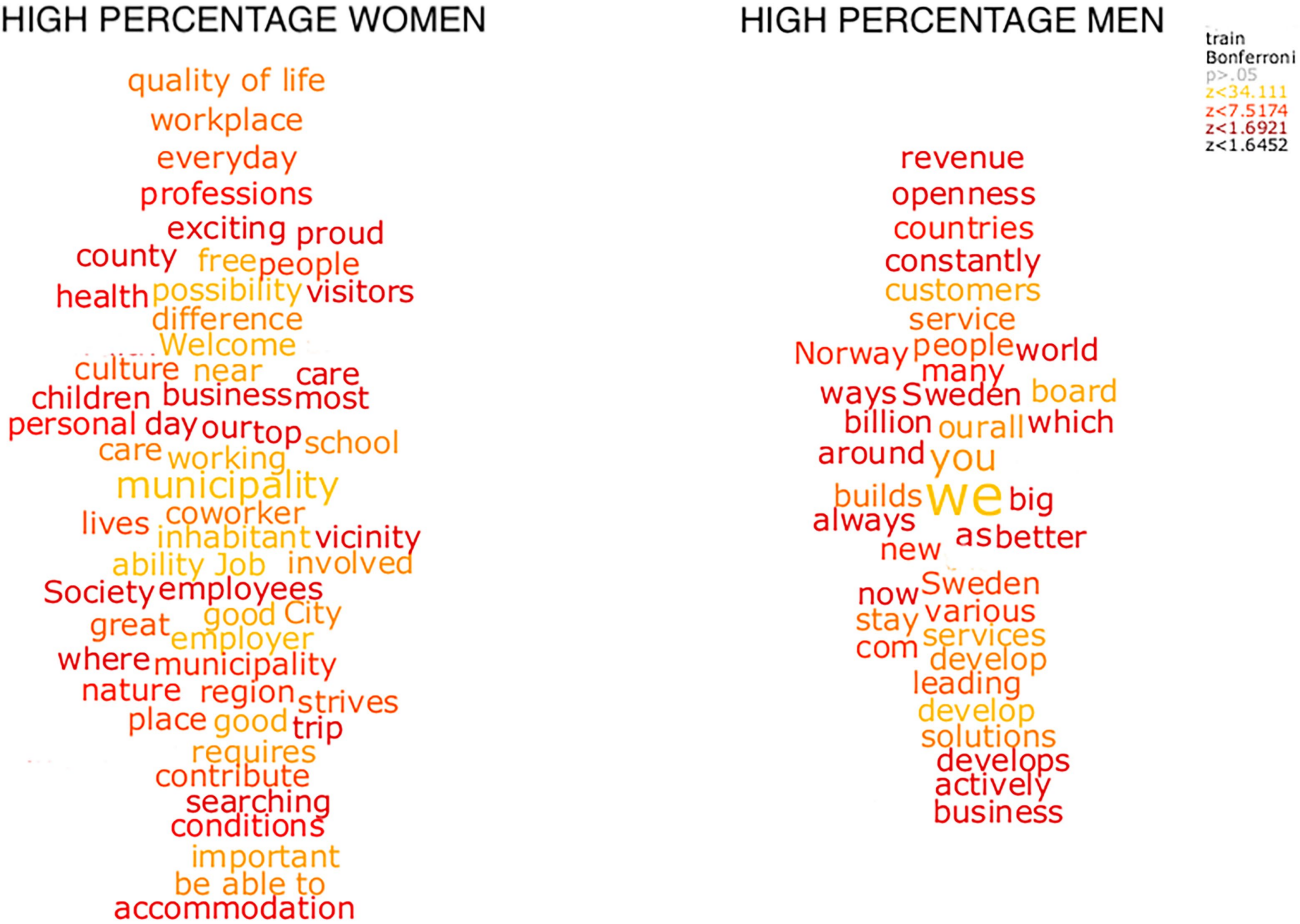
Word clouds depicting words indicative of a high percentage of women and men as significantly tested by training.

The word clouds in Figure 3 represent which words were most indicative of the LinkedIn text set using the semantic *t*-tests by comparing the semantic representations of the LinkedIn texts (text set 1) with text based on the Swedish version of Google n-gram set (text set 2; Google Books Ngram Viewer, 2013). The clouds are based on *z*-values of the semantic *t*-test. The more semantically typical for the LinkedIn data set a word is, the higher the *z*-value. All plotted words were significant following Bonferroni correction for multiple comparisons:

The font sizes in all word clouds represent the frequency of the words in the data set – the bigger the word, the more frequent it is. Colors represent *z*-values of the significance testing of the words (specified in the legend in the upper right corner of Figures 3-5). Translation of the Swedish organizational descriptions was made in Semantic Excel based on Google Translate.^3^ As can be seen, the pronoun *we* stood out as the most commonly used word.

To examine differences in word choice between organizations with a majority of women and men employees, word clouds comparing the two were created. Figures 4, 5 show the words that significantly discriminate between the LinkedIn texts of organizations with high percentages of women and men employees. In Figure 4, significance testing was made by chi-square tests based on the relative word frequency. That is, Figure 4 shows the significant difference in relative frequency (e.g., occurrences per million) of words between data with a majority of women and men employees. Significant words, following Bonferroni correction for multiple comparisons, are shown in these word clouds:

The two clouds contrasted each other, showing different themes. The clouds depicting organizations with a majority of women employees were focused on local and regional services aimed at the citizens, whereas organizations with a majority of men employees were focused on money, competition, customers and both Sweden and other countries.

Figure 5 shows words that significantly discriminate between the ratio of women employees in the organizations in the LinkedIn. Figure 5 was created by the multiple linear regression model described in the Appendix. That is, the regression models were trained using the summarized semantic representations of the LinkedIn texts as input, to predict gender ratio. The words shown in the plots were significant following Bonferroni correction for multiple comparisons.

The word clouds that discriminated between organizations with a majority of women and a majority of men employees were analyzed in terms of agency and communion (Pietraszkiewicz et al., 2018), and by dictionaries used in Linguistic Inquiry and Word Count (LIWC; Pennebaker et al., 2015). In the cloud depicting texts from organizations with a higher percentage of women (to the left), we found words related with *communion* (e.g., care, society, and welcome), *work* (e.g., employee, job, working). In the cloud depicting a higher percentage of men (to the right) we also found words related to communion (e.g., services and people), as well as *numbers/quantity* (e.g., billion, many, and various), and *space* (e.g., world and around). In both clouds we found agency related words (e.g., ability, strives for women dominated industries, and proud, actively for men-dominated industries). All in all, the cloud with a higher percentage of women employees included words reflecting the cooperation and the employer/employee relationship, whereas the cloud with a higher percentage of men employees included words relating to customer relations and numbers.

## Discussion

This study investigated the relation between language in organizational descriptions and the gender ratios of those organizations, based on legal gender (i.e., women/men). How organizations choose to present themselves might reflect whether women or men are the typical employees. The recruitment process is crucial in preventing gender segregation. Because organizational descriptions are likely to influence who identifies with the workplace, and thereby who wants to apply for a job there, a greater understanding of such descriptions and the way they are presented, may help in preventing gender segregation. The current research examined a Swedish context, observing Swedish organizational descriptions on LinkedIn. In contrast to much earlier research that has focused on job adverts, we here argue that a broader focus on organizational descriptions reveals something about how the organization is constituted (Gaucher et al., 2011; Pietraszkiewicz et al., 2018).

We found a strong relationship between language in organizational descriptions and the employee gender ratio of women/men in the organization. This finding is in line with research on job adverts. Pietraszkiewicz et al. (2018) found that job descriptions reflect gender ratio in the profession as measured by percentages of agency and communion words. Gaucher et al. (2011) found that job adverts for men-dominated areas, as compared with women-dominated areas, used words associated with masculine stereotypes. While certain jobs may require communal skills and thus are difficult to entirely change, organizational descriptions could include more balanced language in terms of both agency and communion, thereby increasing a perceived fit with the organization for both women and men. Our results indicate that this may be a neglected area in gender equality work.

The present study further extends prior research on job adverts by using a completely data-driven method examining how organizational descriptions differ between organizations with different gender distributions. Previous research used dictionaries representing feminine/communal and masculine/agentic stereotypical characteristics and word choice (Gaucher et al., 2011; Pietraszkiewicz et al., 2018). Gaucher et al. (2011) used manual coding, and Pietraszkiewicz et al. (2018) used coding based on the methods of LIWC (Pennebaker et al., 2015). The present study was data-driven and thereby inductively examined what words differentiate between texts in organizations with a majority of women and men employees. Descriptions of organizations with a higher percentage of women were characterized by communal words, and also words related with the workplace. In contrast, descriptions of organizations with a higher percentage of men were characterized by an equal balance of agency and communion, but also with more words related with time, space and numbers.

The organizations with a higher percentage of women or a higher percentage of men employees represent different sectors, i.e., women dominate the public sectors (e.g., healthcare and education) and men dominate the private sectors (e.g., industry, transport, and agriculture). Hence, the organizational descriptions could differ due to differences between industries. To further examine this, an additional analysis was conducted. In it, coding for private or public organization was added as a covariate to the multiple linear regression model. The gender ratio was still predicted with a reasonably high Pearson correlation. This indicates that differences in the organizational descriptions to a certain degree do reflect the gender ratios of the organizations, rather than just the different sectors that the organizations belong to. Social role theory (Eagly, 1987) posits that women are assumed to possess communal traits, such as being caring while men are assumed to possess agentic traits such as leading because they are observed in roles that require the corresponding traits. This is also what we found in our analyses. Words reflecting communality and the workplace were found in the organizational descriptions of organizations with a majority of women employees, and words reflecting numbers, space and quantities could be found among the organizational descriptions of organizations with a majority of men employees. While not all words that differed between the women and men dominated organizations completely aligned with the adjectives often presented in social role theoretical research, it is important to keep in mind that such research is focused on descriptions of individuals and therefore are qualitatively different from descriptions of organizations. Still, the important words that were uniquely associated with the women/men-dominated organizations do reflect more relationship-building words among the women-dominated organizations and more goal-oriented words among the men-dominated organizations (Bakan, 1966). Examples of words from the women-dominated organizations were nature, culture, children, care, health, county and quality of life. Such words clearly imply a sort of “connectedness.” Examples of words from the men-dominated organizations were builds, solution, development, actively and revenue. These words are rather focused on goal-achievement. Nonetheless, these words reflect the two overarching dimensions often seen as central in person perception (Trapnell and Paulhus, 2012; Abele and Bruckmüller, 2013), where they are often referred to as morality and ability (Reeder and Brewer, 1979), or in terms of stereotypes, the terms warmth and competence can also be applied (Fiske et al., 2007).

We used two different language models for predicting the gender ratio in the LinkedIn texts. BERT is a modern language based on a transformer deep learning architecture, and we therefore predicted that it would outperform the older and simpler bag-of-word model, LSA. This was also the case, where the BERT model showed somewhat higher accuracy in prediction of the gender ratio compared to the LSA as measured by the Pearson correlation between estimated and empirical measured gender ratios.

The gender ratio of women averaged over all organizations is 64% in our dataset, indicating that there is oversampling of organizations with a higher ratio of women compared to men. It would have been optimal to use a dataset with equal gender ratios. However, to our knowledge the unequal gender ratio does not influence our finding that the gender ratios can be predicted by the text describing the organizations.

## Practical implications

The organizational description can affect potential applicants’ perceptions of the organization as a whole, whereas the job description only affects perceptions of the job. This, in turn, might influence perceived person-organization fit and person-job fit, respectively (Kristof-Brown et al., 2005). The extent to which an applicant is attracted to an organization is the first step in the recruitment process, and here, different messaging strategies are implemented to generate the largest possible pool of qualified applicants (Dineen and Soltis, 2010). In a meta-analysis (Chapman et al., 2005), attraction toward a new job and job pursuit intentions were predicted by perceived person-organization fit, whereas intentions to accept a job offer were predicted by perceived person-job fit. That is, in the early stages of recruitment, person-organization fit predicts critical attitudes of the prospective employee. Recruitments are a central event in maintaining or reducing gender segregation (Lindqvist et al., 2018). Organizational descriptions could influence who identifies with the work place and who wants to apply for a job there (Klysing et al., 2021). The results of the present research indicate different ways of addressing the assumed receiver of the texts, i.e., the presumptive employee, depending on the gender distribution of the organization. If women-dominated and men-dominated industries are described in very dissimilar ways, this could contribute to maintenance of a segregated job market. It is therefore important for organizations to be aware of this in order to prevent qualified candidates from rejecting places of work at an early stage, simply because they do not experience a person-organization fit. In the long run, such practices contribute to uphold gender stereotypes since women will continue to be observed in jobs that require communal skills and men will continue to be observed in jobs that require agentic skills (Eagly, 1987). In addition, studies indicate advantages in having organization with a greater gender balance such as less sexual harassment (Folke and Rickne, 2022), economic growth and innovation (Joecks et al., 2013; Hsieh et al., 2019), and better health (Bryngelson et al., 2011).

In accordance with Swedish law (Discrimination Act, 2008:567), employers should promote an equal gender distribution in different types of work and employee categories, and when the distribution is not more or less equal in a certain type of work or employee category, the employer is to make a special effort when recruiting new employees to attract applicants of the under-represented gender. If organizations want to attract an under-represented category, the lack of fit model (Heilman, 1983) might be of use in helping organizations understand *how* to do it. The model is made up of two constituents: gendered job requirements and gender stereotype characteristics. Minimizing either of these, will also minimize expectations of performance failure and biased recruitments (Heilman and Caleo, 2018). One way for organizations to target the first component is for them to change the perception of their field and of themselves. To achieve this, practices promoting and sustaining views of the organization as gendered must be addressed. Organizational texts must describe the core activity (e.g., medicine or engineering); however, organizational descriptions with a gendered language may help preserve the idea that these organizations require exclusively stereotypically feminine or masculine gendered behavior (e.g., by emphasizing cooperation or competition). Gendered language in job descriptions has been shown to negatively influence both inclination to apply (Gaucher et al., 2011) as well as prospect of being chosen (Horvath et al., 2015) when there is perceived gender incongruence between job and job seeker. When changing the language in organizational or job descriptions, organizations should preferably use a balanced language, associated with both women and men stereotypes, or a more gender-neutral language, so that an intervention does not increase a mismatch among majority members of the organization (Brown and Jacoby-Senghor, 2021) although majority members may pay less attention to smaller shifts in how the organization is framed (Avery, 2003).

## Limitations and future research

The present study had a number of strengths, but was not without limitations. First, as already discussed, the examined organizations were made up of widely different industries, for example commerce, education, public authorities and production/extraction. Certain differences of language in the organizational descriptions were therefore to be expected. This was to a certain degree accounted for using an additional analysis, in which coding for private or public organization was added as a covariate to the multiple linear regression model. The gender ratio could still be predicted with a reasonably high Pearson correlation. However, future research may want to examine more organizations with varying gender distribution within the same industry, for example commerce or finance. This requires information about exact employment demographics from a large number of organizations within the same industry.

Second, only observational data was employed, with no experimental manipulations. Future studies should examine potential applicants’ experiences of the organizational descriptions: their attraction toward the organizations and the likelihood of applying, by using core words from organizational descriptions of organizations with a majority of men employees in descriptions of organizations with a majority of women employees. This would give the opportunity to examine if and how an organization describes itself influences potential candidates. It would also be of interest for future studies to investigate the overlap between an employee’s description of their work place and the official organizational description, as these are promotional texts not necessarily consistent with reality.

The LSA model used in the LinkedIn texts is a generic model of the Swedish language, whereas the BERT model is a multi-lingual model applicable to several languages. Thus, neither of these models have been fined tuned to the specific type of language used in the Swedish LinkedIn texts. Future research may analyze organizational texts with a language model that is fine tuned to the to-be-analyzed text data set. To what extent this will further improve the accuracy in the gender ratio predictions is a topic for future research.

Another avenue for future research would be to test if the gender of the author affects how an organizational description is framed. For example, an organization with a higher female ratio is perhaps more likely to have a woman describe the organization. Thus, the gender of those in charge of making the text could influence how they understand and describe the organization. This would therefore be an interesting variable to look at. However, unfortunately, we do not have access to the gender of the authors. A possibility here is to apply an NLP model that the predicts gender of authors on the LinkedIn texts. However, such an analysis is further complicated by the fact that the texts are presumably written and edited by several people in company with a mix of men and women. Due these complications, we have avoided to make such analysis, although it would be an interesting topic for future research.

Additionally, it would be interesting to examine the relation between language in organizational descriptions and that in job adverts. Previous research has shown that language in job adverts reflect the gender distribution in a profession and vary in word use depending on whether it is a women-or men-dominated job. However, on LinkedIn the organizational descriptions are independent from potential job adverts, as it is a way for the organization as a whole to introduce themselves. Hence, the examined organizations and organizational descriptions in the current study were not linked to any specific job adverts.

Lastly, because the gender distribution of the employees was determined based on legal gender, only the binary genders of women/men were included in the analysis. Given that gender is *not* a binary category (Hyde et al., 2019; Lindqvist et al., 2020), these analyses did hence not include the variation in gender identities. Future research could expand the definition of gender segregation, by also include non-binary identities (i.e., individuals not identifying as women or men). However, this requires other measures of gender distributions, such as self-reported gender identity among the employees.

## Conclusion

In summary, this study showed that organizational descriptions reflect the gender distribution in organizations, based on the legal gender of women/men, with moderate to strong Pearson correlations between observed proportions of women/men in the organizations, and predictions based on training from LinkedIn texts. This suggests that the gender ratio of an organization is in some way linked to the way that organization chooses to describe themselves. The organizational descriptions could thereby communicate subtle signals in regards to what potential candidate is most sought after, and risk not attracting those who are underrepresented in the organization.

## Data availability statement

The original contributions presented in the study are included in the article/Supplementary material, further inquiries can be directed to the corresponding author.

## Author contributions

MG, AL, and ER: conception or design of the work. LS and MG: data collection. LS, SS, and MG: data analysis and interpretation and drafting the article. AL and ER: critical revision of the article. All authors contributed to the article and approved the submitted version.

## Funding

This work was supported by the Swedish Research Council for Health, Working Life and Welfare (grant no. 2017–00414).

## Conflict of interest

The authors declare that the research was conducted in the absence of any commercial or financial relationships that could be construed as a potential conflict of interest.

## Publisher’s note

All claims expressed in this article are solely those of the authors and do not necessarily represent those of their affiliated organizations, or those of the publisher, the editors and the reviewers. Any product that may be evaluated in this article, or claim that may be made by its manufacturer, is not guaranteed or endorsed by the publisher.

## Footnotes

^1^ https://www.scb.se/

^2^ https://github.com/sverkersikstrom/semanticCode

^3^ https://translate.google.com/
