# Supplementary material for: Language and gender: Computerized text analyses predict gender ratios from organizational descriptions
Source: Front Psychol. 2023 Jan 9;13:1020614. doi: 10.3389/fpsyg.2022.1020614 (PMC9869037; doi:10.3389/fpsyg.2022.1020614)
Supplement: Supplementary file 1 [file Data_Sheet_1.docx]

**Appendix**

**Predicting gender ratios from text data**

**LSA**

LSA is a bag-of-word model that creates semantic representations of words, but cannot capture the grammatical structure of the data. According to LSA, information about the meaning of a word is generated from the contexts that the word appears in, and this information can be used to quantify semantic meaning in a high dimensional semantic space (Landauer and Dumais, 1997). LSA creates a *semantic space*, which is a matrix where words are represented in the rows, and the columns represent a large number of semantic dimensions. Semantic spaces are created on text data. The semantic spaces in SemanticExcel are built from “Google Books Ngram Viewer” (2013). This choice was made because it is, to our knowledge, the largest public accessible database to the Swedish language. This database is made up of huge amounts of diverse texts (100 billion words in Swedish) from several million books and texts from the Internet (Brants and Franz, 2009; Michel et al., 2011).

We used the Swedish semantic space that is described in detail in Kjell et al. (2018), however, here we provide a brief overview of the method. The semantic spaces are based on the Swedish version of Google 5-gram, i.e., a sequence of five words (see first text box in Figure A1). The choice of 5-gram was based on the fact that this is the largest N-gram available on Google N-gram, and is therefore the largest available context size in this dataset. The NLP literature often suggests that high frequency words should be removed as it carries little information of the content of the texts. However, here the focus is related to social psychology where a prominent view is that function words (which often includes very high frequency words, for example pronouns) carry essential information. For example, Chung and Pennebaker (2007) suggested that function words are essential for understanding phenomena in social psychology. Due to this reason, high frequency words are maintained in the semantic representation. Based on the 5-gram context, a word-by-word frequency co-occurrence table is created, in which the 120 000 most commonly used words from the 5-gram contexts are listed in the rows and the 50 000 most common words in the columns (see second text box in Figure A1). The number of times these words co-occur within the 5-gram word contexts is then counted. For example, the number in the cell where *employee* and *work* meet, reflects how many times these words co-occur in a 5-gram context. In the next step, the semantic dimensions are created using singular value decompositions, SVD (Golub and Kahan, 1965) – a kind of factor analysis aiming to maintain as much information from the original matrix in as few dimensions as possible. That means reducing the large number of columns to a smaller number of semantic dimensions.

*****Figure A1*****

The resulting matrix is the semantic space (see third text box in Figure A1). In this space, the rows represent words and the columns represent dimensions (e.g., Landauer et al., 1998). Each dimension captures semantic features of the words. The value in each cell represents the strength of a semantic feature for a given word. In this way, the semantic space maps words to numbers. The number of dimensions used in the space was optimized to 256 based on a Swedish synonym test, where less, or more, dimensions produced poorer performance on this test (for details see Kjell et al., 2018). The length of each vector representing a word in the semantic representation is normalized to the length of one.

The rows in the semantic space are vectors, i.e., ordered sets of numerical values, which describe how words semantically relate to each other. In this way, each vector represents the *semantic representation* of a word in the space (see third text box in Figure A1). A semantic representation can either represent a given word, for example *employee*, or a text sequence, for example a description from LinkedIn. That is, it is possible to create *one* vector in the semantic space for a whole text, rather than just for one word. This is achieved by adding up the semantic representations of all words in a text. For example, paragraphs, sentences, or a whole book can be aggregated to a single vector in the space, where the length of the resulting vector is normalized to the length of one.

In the present study, each organizational description from LinkedIn was aggregated to a semantic representation. The semantic representations from individual words of each of the LinkedIn texts were added together, semantic dimension by semantic dimension, to represent one single semantic representation. As a result, each organizational description from LinkedIn was represented as a vector in the semantic space. The aggregated semantic representation was normalized to the length of one by dividing each semantic dimension value with the length of the semantic representation. This simplifies computations of semantic similarities.

**BERT**

For the BERT model, we used the multi-lingual cased version (bert-base-multilingual-cased), which has been pre-trained on the top 104 languages with the largest Wikipedia (i.e., including Swedish) using a masked language modeling (MLM) objective (for details on this training see Devlin et al., 2019). This model is case sensitive, so it makes a difference between stockholm and Stockholm. Thus, using this BERT model allowed us to input Swedish text to BERT, without the need for translating it to English. The representation on the last layer (N = 12) of the BERT model was used. Finally, we used singular value decomposition, SVD (Golub and Kahan, 1965), as a data compression algorithm to rearrange the dimensions so that the first dimensions contain the most information of the representation. This was needed as we wanted to compare with the LSA model, where the first dimensions contain the most important information.

**Results for the BERT model**

The Pearson correlation between the observed and predicted binary employee gender ratio of the organizations was significantly larger than zero for the BERT model (*r* = .69, p < .001, r^2^ = 48%, Mean Absolute Error = 11.6%). The squared error of the BERT model was significantly lower than the squared error of the LSA model (paired two-sided t-test: t(408) = -2.0, p = 0.046 < .05) After using the binary variable coding for private/public organization as a covariate to the multiple linear regression model based on the LSA representations, the results still showed that the gender ratio still could be predicted (*r* = .57, , r^2^ = 32%, p < .001, Mean Absolute Error = 11.3%).

**Predicting gender ratios using multiple linear regression**

Semantic representations can be used as predictors (*x*) in linear regressions (*y* = *w***x*) to predict observed numerical values (*y*), where the semantic representations, i.e., defining the relationship between the numerical values and the words (*w*) can be used to make *semantic estimates* ($\hat{y}$), for a specified text*.* In this way, it is possible to test the significance of a relationship between texts and observed numerical values (e.g., gender ratios) by correlating the estimated and the observed values.

In the present study, we investigated the relationship between 409 unique organizational descriptions from LinkedIn, and their percentage employee gender ratio. Regression models were trained using the summarized semantic representations of the LinkedIn texts as input, to predict the gender ratio. A leave-n-out *cross-validation* procedure (see e.g., Yarkoni and Westfall, 2017) was used to avoid including known information in the evaluation of the regression models. The leave-n-out cross-validation randomly divides the data (i.e., the LinkedIn texts accompanied by observed gender ratios) into a training set and a test set. The training set consists of 90% of the data, and the test set of 10% of the data (i.e., a leave-10%-out cross-validation technique). The regression model is thus trained using 90% of the data, when estimating the parameters (i.e., the coefficients defining the relationship between the text and the observed numerical value). The number of dimensions used in each training was optimized by picking the number of dimensions that provided the highest Pearson correlation between empirical and predicted data in the training data set, where only the first N dimensions were used.

For the LSA model, the number of dimensions was optimized to N = 42 (SD = 5). For the BERT mode, the number of dimensions was optimized to N = 19 (SD = 0). The regression model with its estimated parameters is then applied on the test set (i.e., 10% of the data) to test its validity. This cross-validation is repeated in ten steps using non-overlapping test sets, until all observed values have been given a predicted value. Furthermore, to avoid overfitting due to an excessive number of predictor variables, the number of dimensions were optimized in each cross-validation, choosing the number of dimensions that provided the highest Pearson correlation between the predicted and empirical value.

**Figure A1.** Conceptual illustration of the creation of a semantic space and its semantic representations. Adapted from (Sikström and Garcia, 2020).

**References**

Brants, T., and Franz, A. (2009) *Web 1T 5-gram, 10 European Languages Version 1 LDC2009T25*. Philadelphia, PA: Linguistic Data Consortium

Devlin, J., Chang, M. W., Lee, K., and Toutanova, K. (2019). *BERT: Pre-training of Deep Bidirectional Transformers for Language Understanding. In: Proceedings of the 2019 Conference of the North American Chapter of the Association for Computational Linguistics: Human Language Technologies, Volume 1 (Long and Short Papers).* Minneapolis, MN: Association for Computational Linguistics, 4171–4186. doi:10.18653/v1/N19-1423.

Chung, C., and Pennebaker, J. (2007). “The psychological functions of function words,” in *Social Communication*, ed K. Fiedler (New York, NY: Psychology Press), 343–359.

Golub, G. H. and Kahan, W. M. (1965). Calculating the singular values and pseudo-inverse of a matrix. *J Soc Ind Appl Math.* 2, 205-224. doi:10.1137/0702016

Google Books Ngram Viewer (2013) [Website] Available online at: https://books.google.com/ngrams

Kjell, O., Kjell, K., Garcia, D., and Sikström, S. (2018). Semantic measures: Using natural language processing to measure, differentiate, and describe psychological constructs. *Psychol Methods.* 24, 92-115. doi:10.1037/met0000191

Landauer, T. K., and Dumais, S. T. (1997). A solution to Plato's problem: The latent semantic analysis theory of acquisition, induction, and representation of knowledge. *Psychol Rev.* 104, 211-240. doi:10.1037/0033-295x.104.2.211

Landauer, T. K., Foltz, P. W., and Laham, D. (1998). An introduction to latent semantic analysis. *Discourse Process.* 25, 259-284. doi:10.1080/01638539809545028

Michel, J. B., Shen, Y. K., Aiden, A. P., Veres, A., Gray, M. K., Pickett, J., Hoiberg, D., Clancy, D., Norvig, P., Orwant, J., Pinker, S., Nowak, M., and Aiden, E. L. (2011). Quantitative analysis of culture using millions of digitized books. *Science,* 331, 176–182. doi:10.1126/science.1199644

Sikström, S., and Garcia, D. (2020). S*tatistical Semantics – Methods and Applications*. doi:10.1007/978-3-030-37250-7

Yarkoni, T., and Westfall, J. (2017). Choosing prediction over explanation in psychology: lessons from machine learning. *Perspect Psychol Sci.* 12, 1100–1122. doi:10.1177/1745691617693393
